# Plasmonic Spherical Heterodimers: Reversal of Optical Binding Force Based on the Forced Breaking of Symmetry

**DOI:** 10.1038/s41598-018-21498-4

**Published:** 2018-02-16

**Authors:** M. R. C. Mahdy, Md. Danesh, Tianhang Zhang, Weiqiang Ding, Hamim Mahmud Rivy, Ariful Bari Chowdhury, M. Q. Mehmood

**Affiliations:** 1 Department of Electrical & Computer Engineering, North South University, Bashundhara, Dhaka 1229 Bangladesh; 2 Department of Electrical and Computer Engineering, National University of Singapore, 4 Engineering Drive 3, 117583 Singapore, Singapore; 3 Pi Labs Bangladesh Ltd., ARA Bhaban, 39, Kazi Nazrul Islam Avenue, Kawran Bazar, Dhaka Bangladesh; 4 NUS Graduate School for Integrative Sciences and Engineering, National University of Singapore, 28 Medical Drive, Singapore, 117456 Singapore; 5 Department of Physics, Harbin Institute of Technology, Harbin, 150001 People’s Republic of China; 6 Department of Public Health, North South University, Bashundhara, Dhaka 1229 Bangladesh; 7 Department of Electrical Engineering, Information Technology University of the Punjab, 54000 Lahore, Pakistan

## Abstract

The stimulating connection between the reversal of near-field plasmonic binding force and the role of symmetry-breaking has not been investigated comprehensively in the literature. In this work, the symmetry of spherical plasmonic heterodimer-setup is broken forcefully by shining the light from a specific side of the set-up instead of impinging it from the top. We demonstrate that for the forced symmetry-broken spherical heterodimer-configurations: reversal of lateral and longitudinal near-field binding force follow completely distinct mechanisms. Interestingly, the reversal of longitudinal binding force can be easily controlled either by changing the direction of light propagation or by varying their relative orientation. This simple process of controlling binding force may open a novel generic way of optical manipulation even with the heterodimers of other shapes. Though it is commonly believed that the reversal of near-field plasmonic binding force should naturally occur for the presence of bonding and anti-bonding modes or at least for the Fano resonance (and plasmonic forces mostly arise from the surface force), our study based on Lorentz-force dynamics suggests notably opposite proposals for the aforementioned cases. Observations in this article can be very useful for improved sensors, particle clustering and aggregation.

## Introduction

**Figure 1:**
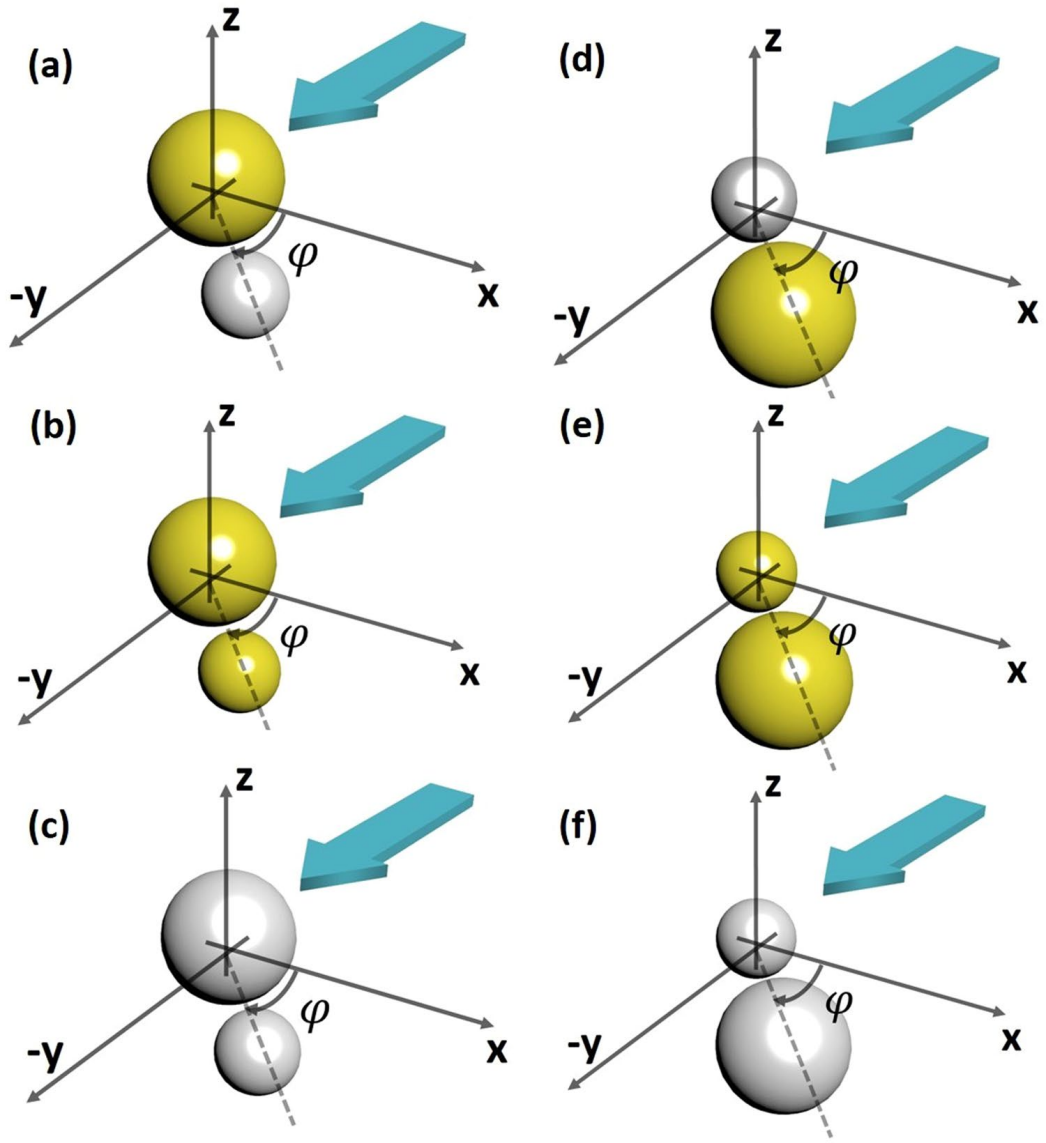
Two particles of radii 100 and 50 nm are placed with inter particle distance from surface to surface ‘*d*’; *d* = 20 nm throughout the article. One particle centered at (0,0,0) and the other centered at $(R\,\cos \,\phi ,-R\,\sin \,\phi ,0)$ with *R* = *d* + *r*_1_ + *r*_2_ = 170 nm the center-to-center distance of the two object. The angular displacement is ‘*φ*’ which is considered 0 degree when the dimers are on-axis in *x* direction. And the angular displacement is considered + 90 degree [end fire configuration] when the dimers are on-axis in −*y* direction. Two different polarized light sources are applied propagating towards −*y* direction [in order to break the symmetry forcefully; light is shined from a specific side instead of the top side of the dimers]: (i) For parallel polarization: *x-*polarized plane wave ${E}_{x}={E}_{0}{e}^{i\beta y}\,$(ii) For perpendicular polarization: *z-*polarized plane wave *E*_*z*_ = *E*_0_*e*^*iβy*^. Yellow color represents Au and Silver color represents Ag: (**a**) Au-Ag (**b**) Au-Au (**c**) Ag-Ag (**d**) Ag-Au (**e**) Au-Au and (**f**) Ag-Ag.

Fano resonances, super-scattering and plasmonic hybridization in nanostructures^1–4^ have received substantial attention in the area of plasmonics. The promising applications of plasmonic hybridization, super-scattering and Fano resonances have been investigated in improved sensitivity of the resonance^5^, bio sensing^6^, surface-enhanced Raman scattering^7,8^, photonic propagation and wave guiding^9,10^, plasmon-induced transparency^11^ to super scattering^12^ and many others^13,14^. In contrast, much less attention is dedicated on near field optical force due to Fano resonance and plasmonic hybridization; especially for plasmonic dimers^1,2,15–19^. It is important to note that heterodimers show remarkable properties such as Fano resonances^1,17^, avoided crossing behavior^1^, optical nanodiode effect^1^ and so on. But the behavior of near field optical force for such heterodimers has not been studied in detail. So far only two works^20,21^, as far as of our knowledge, have studied the behavior of binding force for *on-axis spherical heterodimers*. Though the behavior and reversal of near field optical binding force for spherical plasmonic on-axis *homodimers*^22–26^ (due to bonding and anti-bonding modes without any substrate) have been studied comprehensively, such detailed investigations lack for on-axis^20,21^ and *off-axis spherical heterodimers*. Here off-axis means end-fire^20^ and nearly end-fire configuration [cf. Fig. 1 when the rotation angle, *φ*, of the particle is between around 70 to 110 degrees from positive *x* axis]. Most importantly, answers of several important questions regarding the near field plasmonic binding force are still fully unknown; such as:

- Based on Fano resonance, induced from heterodimer set-ups, the reversal of near field optical binding force has been reported in^27^ and^28^ for nanorod structures^27^ and for disk along with a *ring* structure^28^. The answer of the question, whether such Fano resonance (raised from heterodimer interaction) is a universal process of achieving near field binding force reversal or not, is still unknown.
- A homodimer structure under longitudinal polarization supports only bonding plasmon mode (*σ*). Likewise, only an anti-bonding π*-mode is observed in a homodimer under transverse polarization^2^. It has been discussed in detail in^24^ that: if two plasmonic objects approach each other, the single-object plasmons hybridize and split into attractive bonding modes and repulsive antibonding modes, which ultimately evolve into an attractive band and a repulsive band. In contrast to the plasmonic homodimers^2^, a plasmonic “heterodimer” structure supports both bonding and antibonding plasmon modes at the same time due to its ‘naturally’ broken symmetry as demonstrated in^2^. As a result, it is expected that binding force reversal should occur for such heterodimer structures due to the presence of bonding and antibonding modes. But does it, reversal of near field binding force for spherical plasmonic heterodimers, really happen all the times?

In this work, (a) at first we have demonstrated that Fano resonance^1,4^ does not contribute to binding force reversal for spherical plasmonic heterodimers. As a result, we conclude that Fano resonance may not be considered as a universal process of the reversal of near field binding force. (b) Later, it is also demonstrated that even with the presence of both bonding and anti-bonding modes^24^, reversal of binding force may not occur for spherical plasmonic heterodimers. So, if both (a) and (b) do not favour to achieve the reversal of binding force for spherical plasmonic heterodimers, is there any alternative way to achieve and control it?

**Figure 2:**
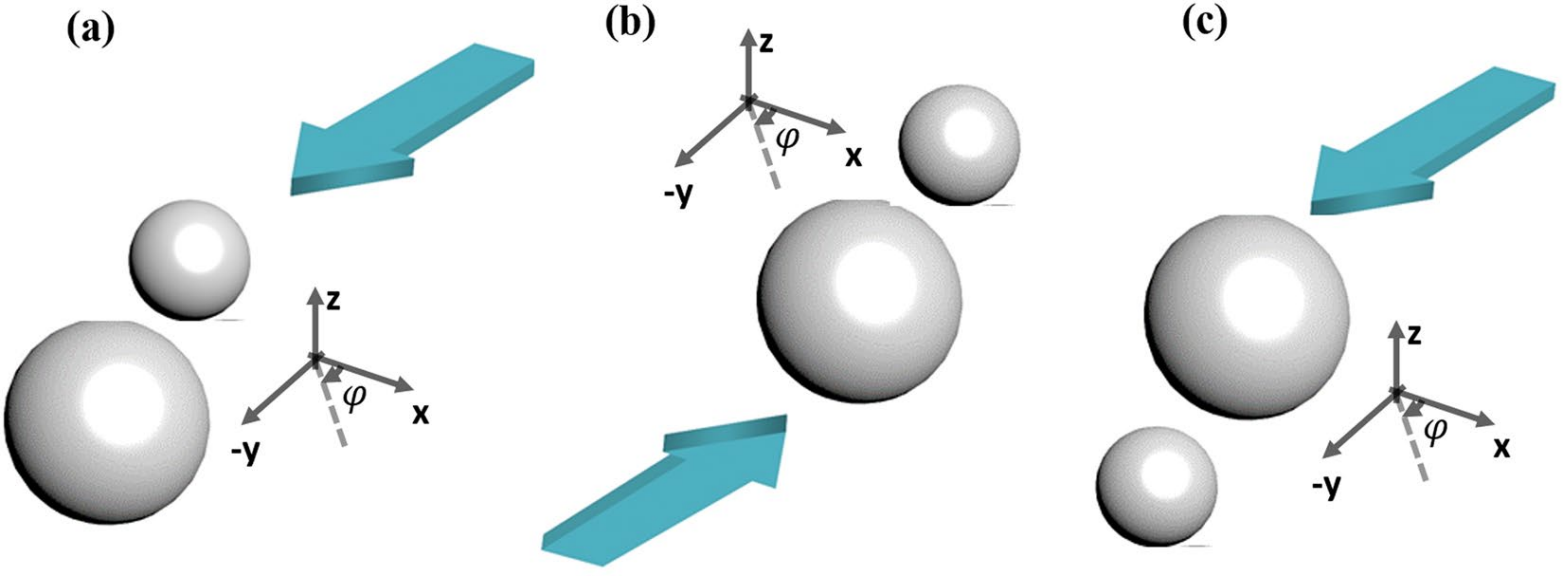
(**a**) The configuration of rotating the bigger object by keeping the smaller object fixed as discussed in previous figure (light is always propagating towards ‘−y’ direction). At higher wavelength regions, near field binding force is always found *repulsive* for this configuration after the antibonding resonance mode. (**b**) For the configuration of Fig. (**a**), the change of the direction of propagating light manually by bringing the light source from one side of the dimers to another side (light is propagating towards ‘+y’ direction). All the times, near field binding force is found *attractive* when the propagating light is perturbed by the bigger object at first. So, combining (**a**) and (**b**), It will be easily possible to observe the mutual repulsion and attraction of all the heterodimer sets just due to the automatic change of the relative dimer position of smaller and bigger objects at higher wavelength regions after the antibonding resonance mode (discussed in main text before conclusion). (**c**) Another alternative way: by changing the relative orientation of the heterodimers of (**a**) manually (not light propagation direction), it is also possible to observe reversal of binding force at higher wavelength regions after the antibonding resonance mode.

To answer the aforementioned questions, we have introduced the idea of ‘Forced breaking of symmetry’ of heterodimers. In this article, ‘Forced breaking of symmetry’ means shining the light from a particular side of the dimers instead of the top (or bottom) side as shown in Fig. 1 [also discussed later in detail]. Most importantly, as the main final proposal of this article, we have demonstrated that the reversal of near field longitudinal binding force can be easily controlled based on the ‘forced’ symmetry breaking just by changing the relative orientation of the heterodimer set-up in two different ways as shown in Fig. 2. Such simple control may not be possible with the spherical homo-dimers^22–26^ to achieve the all-optical clustering and aggregation.

Another important fact is that: though Lorentz force analysis has been applied previously in^29–33^ to understand several different purposes; such a fundamental analysis is rare to understand the plasmonic effects and plasmonic binding force. It is commonly believed that plasmonic forces mostly arise from the surface force/polarization induced charges^34,35^. But our study based on Lorentz force suggests a notably different proposal especially for the off-axis (near end fire) heterodimers.

Table 1 *of this article* (*given below*) *represents a very short overview of our overall investigation throughout this article, which suggests that the reversal mechanism of lateral and longitudinal binding force follow fully different mechanisms*. However, Table 1 represents only few possible cases, which ultimately lead us to the possible final conclusion/main proposal of this article to control longitudinal binding force based on ‘forced’ symmetry breaking:

**Table 1:** An overview on the binding force for spherical heterodimers (only few possible cases).

Hetero-dimer Set	Number in Fig. 1	On- axis [*ȹ* = 0]	Off- Axis [*ȹ* = 70 to 110 deg.]	Polar. || = Parallel ⊥ = Perpend.	Binding Force reversal: (i)Lateral (ii)Longitudinal	Comment: (a) Inter-particle edge to edge gap, d, is always fixed 20 nm. (b) Heterodimer radii are fixed: 50 nm and 100 nm.
Ag-Au	(a) [=(d)]	Yes	No	⊥	(i)Yes	Lateral near field binding force reverses only for perpendicular polarization. For higher wavelength region: Reversal of force occurs due to zero surface and bulk Lorentz force at a specific wavelength near bonding (attractive force) resonance. For lower wavelength region: such force reversal can be recognized from the reversal of electric dipole moment of the smaller object. In fact, such reversals (repulsive to attractive) occur due to induced electric resonance near the bonding resonance mode. Notably, for the parallel polarization, the presence of Fano resonance cannot help to reverse the binding force.
Au-Au	(b) [=(e)]	Yes	No	⊥	(i)Yes	
Ag-Ag	(c) [=(f)]	Yes	No	⊥	(i)Yes	
Ag-Au	(a) [=(d)]	Yes	No	||	(i)No	
Au-Au	(b) [=(e)]	Yes	No	||	(i)No	
Ag-Ag	(c) [=(f)]	Yes	No	||	(i)No	
Ag-Au	(a)	No	Yes	⊥ and ||	(ii) No	Longitudinal near field binding force reverses [for only Ag-Au and Ag-Ag case] only when the bigger particle rotates and the light is perturbed by the fixed smaller object at first. This reversal occurs due to the constructive dipole-quadrupole resonance and due to the dominance of the bulk Lorentz force. However, for all heterodimer sets attractive and repulsive force can be very easily controlled by changing the light propagation direction or changing the relative orientation of the dimers. Such control is not possible with the spherical homo-dimers.
Au-Au	(b)	No	Yes	⊥ and ||	(ii) No	
Ag-Ag	(c)	No	Yes	⊥ and ||	(ii) No	
Ag-Au	(d)	No	Yes	⊥ and ||	(ii) Yes	
Au-Au	(e)	No	Yes	⊥ and ||	(ii) No	
Ag-Ag	(f)	No	Yes	⊥ and ||	(ii) Yes	

‘Reversal of optical longitudinal binding force can be easily controlled by controlling the relative orientation (in two distinct ways) of the spherical heterodimers as shown in detail in Fig. 2 (and it will be discussed again in forthcoming last section just before the Conclusion section).’ To achieve the all-optical clustering and aggregation, this work may also open a novel track; such as: whether such controlled reversal of near field longitudinal optical binding force is possible for other shaped heterodimers or not based on the ‘forced’ braking of symmetry.

## Results and Discussion

We specify that throughout this paper we refer to ‘exterior’ or ‘outside’ forces as those evaluated outside the volume of the macroscopic particles, while ‘interior’ or ‘inside’ refer to those quantities inside this object volume. To consider the realistic effects, we have done all the numerical calculations /full wave simulations in three dimensional (3D) structures using Lumerical FDTD software^36^ commercial software [and also verified by using COMSOL MULTI PHYSICS software^37^].

The proposed simplest set-up is illustrated in Fig. 1. The Gold and Silver particles are placed near to each other. The real and imaginary part of the permittivity of Gold and Silver are taken from the standard CRC and Palik data^36,38^. Inter particle distance is ‘d’. The source is a simple *x or z -*polarized (for parallel and perpendicular polarizations respectively; cf. Fig. 1) plane wave $E={E}_{0}{e}^{i\beta y}\,$ propagating in ‘−*y’* direction; where ${E}_{0}=\,1\,v/m$. This set-up is a ‘forced’ symmetry broken system, which later plays a vital role for the force reversal. If the heterodimer set-up is shined from the top, such ‘forced’ symmetry breaking is not possible. The ‘outside optical force’^39,40^ is calculated by the integration of time averaged Minkowski^20–28,39,40^ stress tensor at r = *a*^+^ employing the background fields of the scatterer of radius *a*:

1 $$\begin{array}{c}\langle {{\boldsymbol{F}}}_{{\rm{Total}}}^{{\rm{Out}}}\rangle =\oint \langle {\bar{\bar{{\boldsymbol{T}}}}}^{{\rm{out}}}\rangle \cdot d{\boldsymbol{s}}\\ \langle {\bar{\bar{{\boldsymbol{T}}}}}^{{\rm{out}}}\rangle =\frac{1}{2}{\rm{Re}}\,[{{\boldsymbol{D}}}_{{\rm{out}}}{{\boldsymbol{E}}}_{{\rm{out}}}^{\ast }+{{\boldsymbol{B}}}_{{\rm{out}}}{{\boldsymbol{H}}}_{{\rm{out}}}^{\ast }-\frac{1}{2}\bar{\bar{{\bf{I}}}}({{\boldsymbol{E}}}_{{\rm{out}}}^{\ast }\cdot {{\boldsymbol{D}}}_{{\rm{out}}}+{{\boldsymbol{H}}}_{{\rm{out}}}^{\ast }\cdot {{\boldsymbol{B}}}_{{\rm{out}}})].\end{array}$$

Where ‘out’ represents the exterior total field of the scatterer; ***E***,***D***, ***H*** and ***B*** are the electric field, displacement vector, magnetic field and induction vectors respectively, 〈〉 represents the time average and $\overline{\overline{I}}$ is the unity tensor.

On the other hand, based on the Lorentz force, the total force (surface force and the bulk force^29–33^) can be written as:

2 $$\langle {{\boldsymbol{F}}}_{{\rm{Total}}}\rangle =\langle {{\boldsymbol{F}}}_{{\rm{Volume}}}\rangle =\langle {{\boldsymbol{F}}}_{{\rm{Bulk}}}\rangle +\langle {{\boldsymbol{F}}}_{{\rm{Surf}}}\rangle =\int \langle {{\boldsymbol{f}}}_{{\rm{Bulk}}}\rangle dv+\int \langle {{\boldsymbol{f}}}_{{\rm{Surface}}}\rangle ds$$

where

3 $$\begin{array}{rcl}\langle {{\boldsymbol{f}}}_{{\rm{Surface}}}\rangle  & = & {\sigma }_{e}{{\boldsymbol{E}}}_{avg}^{\ast }+{\sigma }_{m}{{\boldsymbol{H}}}_{avg}^{\ast }\\  & = & \{{\varepsilon }_{{\rm{0}}}({{\boldsymbol{E}}}_{{\rm{out}}}-{{\boldsymbol{E}}}_{{\rm{in}}})\cdot \hat{{\boldsymbol{n}}}\}{(\frac{{{\boldsymbol{E}}}_{{\rm{out}}}+{{\boldsymbol{E}}}_{{\rm{in}}}}{2})}^{\ast }+\{{\mu }_{0}({{\boldsymbol{H}}}_{{\rm{out}}}-{{\boldsymbol{H}}}_{{\rm{in}}})\cdot \hat{{\boldsymbol{n}}}\}{(\frac{{{\boldsymbol{H}}}_{{\rm{out}}}+{{\boldsymbol{H}}}_{{\rm{in}}}}{2})}^{\ast }\end{array},$$

4 $$\langle {{\boldsymbol{f}}}_{{\rm{Bulk}}}\rangle =\frac{1}{2}{\rm{Re}}\,[{\varepsilon }_{0}(\nabla \cdot {{\boldsymbol{E}}}_{{\rm{in}}}){{\boldsymbol{E}}}_{{\rm{in}}}^{\ast }+{\mu }_{0}(\nabla \cdot {\boldsymbol{{\rm H}}}){{\boldsymbol{H}}}_{{\rm{in}}}^{\ast }]-\frac{1}{2}{\rm{Re}}[i\omega ({\varepsilon }_{s}-{\varepsilon }_{0})\{{{\boldsymbol{E}}}_{{\rm{in}}}\times {{\boldsymbol{B}}}_{{\rm{in}}}^{\ast }\}+i\omega ({\mu }_{s}-{\mu }_{0})\{{{\boldsymbol{D}}}_{{\rm{in}}}^{\ast }\times {{\boldsymbol{H}}}_{{\rm{in}}}\}]$$

***f***_Surface_ is the surface force density (the force which is felt by the bound electric and magnetic surface charges of a scatterer), which is calculated just at the boundary of a scatterer^29–33^. ***f***_Bulk_ is the bulk force density, which is calculated from the interior of the scatterer by employing the inside field^29–33^. ‘in’ represents the interior fields of the scatterer; ‘avg’ represents the average of the field. *σ*_*e*_ and *σ*_*m*_ are the bound electric and magnetic surface charge densities of the scatterer respectively. *ε*_*s*_ is permittivity and *μ*_*s*_ is permeability of the scatterer. The unit vector $\hat{n}$ is an outward pointing normal to the surface. As long as we know, the Lorentz force dynamics of plasmonic particles and especially heterodimers have not been discussed previously. It is notable that the ‘external dipolar force’^39,40^ (which has also been described as Lorentz force in^20^) is quite different than the Lorentz force defined in our Eqs (2)–(4). Even if the quasi static analysis (i.e. dipolar force^41,42^) leads to wrong conclusion (for example- in refs^20,43–45^); the agreement of Lorentz volume force^29–33,46^ and external ST method^39,40,47–51^ based on full electrodynamic analysis, which is considered for all the force calculations in this article, should lead to the consistent result for realistic experiments. The difference of the scattering part [cf. Eq. (4)] or bulk part of the total Lorentz force on a plasmonic object should describe the relative bulk force experienced by the optical molecule:

5 $${\rm{Del}}\,{{\rm{F}}}_{{\rm{Bulk}}}=\int [\langle {{\boldsymbol{f}}}_{{\rm{Bulk}}(B)}\rangle d{v}_{(B)}]-\int [\langle {{\boldsymbol{f}}}_{{\rm{Bulk}}(S)}\rangle d{v}_{(S)}]$$

Here; subscript (B) and (S) represent: bigger object and smaller object respectively. At the same time the difference of the gradient part [which originates from induced surface charges; cf. Eq. (3)] of the total Lorentz force on a plasmonic object should describe the relative surface force experienced by the optical molecule:

6 $${\rm{Del}}\,{{\rm{F}}}_{\mathrm{Surf}(x)}=\int [\langle {{\boldsymbol{f}}}_{\mathrm{Surface}({B})}\rangle d{s}_{(B)}]-[\int \langle {{\boldsymbol{f}}}_{\mathrm{Surface}(S)}\rangle d{s}_{(S)}]$$

It should be noted that: ${F}_{Bind}=({F}_{B}-{F}_{S})={\rm{Del}}\,{{\rm{F}}}_{{\rm{Bulk}}}+{\rm{Del}}\,{{\rm{F}}}_{{\rm{Surf}}}$.

### Lateral binding force: On-Axis Spherical Heterodimers

Behavior of optical binding force for on-axis spherical heterodimers has been studied in^20^ considering the inter particle edge to edge gap of only 2 nm. In addition, the size of the spherical objects has been considered only 10 nm and another one maximum 40 nm in^20^. However, we have observed that if the inter particle gap is increased (i.e. 20 nm instead of 2 nm), the reversal of optical binding force dies out for both polarizations of light. Still by optimizing the size of the heterodimers a more generic way of binding force reversal has been demonstrated in the next sub-sections.

#### Parallel Polarization: No reversal of lateral binding force for Au-Ag, Au-Au and Ag-Ag on-axis heterodimers

For Ag-Au, Au-Au and Ag-Ag heterodimer configuration, the lateral binding force, F_Bind(x)_ = (F_B(x)_ − F_S(x)_), reversal does not occur for the light polarized parallel to the dimer axis [cf. Fig. 2s in Supplement S2(a) and Fig. 5s(c) in supplement S3]. Here F_B(x)_ and F_S(x)_ are the + *x*-directed time averaged force on big and small particle respectively. According to our detail discussion in Supplement S2(a), the important conclusion is that although reversal of lateral optical binding force occurs for nano rods or other shapes due to Fano resonance^27,28^, Fano resonance is not a generic way/reason of the reversal of optical binding force; i.e. for spherical on-axis heterodimers.

#### Perpendicular Polarization: Reversal of lateral binding force for Au-Ag, Au-Au and Ag-Ag on-axis heterodimers

At first, we consider two on-axis Ag-Au, Au-Au and Ag-Ag particles of 100 and 50 nm with inter particle distance (edge to edge distance) of 20 nm [cf. Fig. 1(a,b,c)] and perpendicular polarized light.

1. It is observed that near the bonding resonance mode reversal of the optical binding force (negative to positive) occurs at the wavelength of 646 nm [cf. Fig. 3(a,b) for Ag-Au and Fig. 3(e,f) for Au-Au]. Reversal of optical binding force occurs at that specific wavelength mainly due to the individual zero surface (${\rm{Del}}\,{{\rm{F}}}_{{\rm{Surf}}(x)}=0$) and bulk (${\rm{Del}}\,{{\rm{F}}}_{{\rm{Bulk}}(x)}=0$) Lorentz force on optical molecule [cf. Fig. 3(c,d) for Ag-Au and Fig. 3(g,h) for Au-Au; detail analysis is given in Supplement S2(b)]. A sudden change is observed in the phase of steady current as well as in the surface charge distribution in Fig. 3s in Supplement S2(b). We have discussed more detail on this topic in Supplement S2(b).

**Figure 3:**
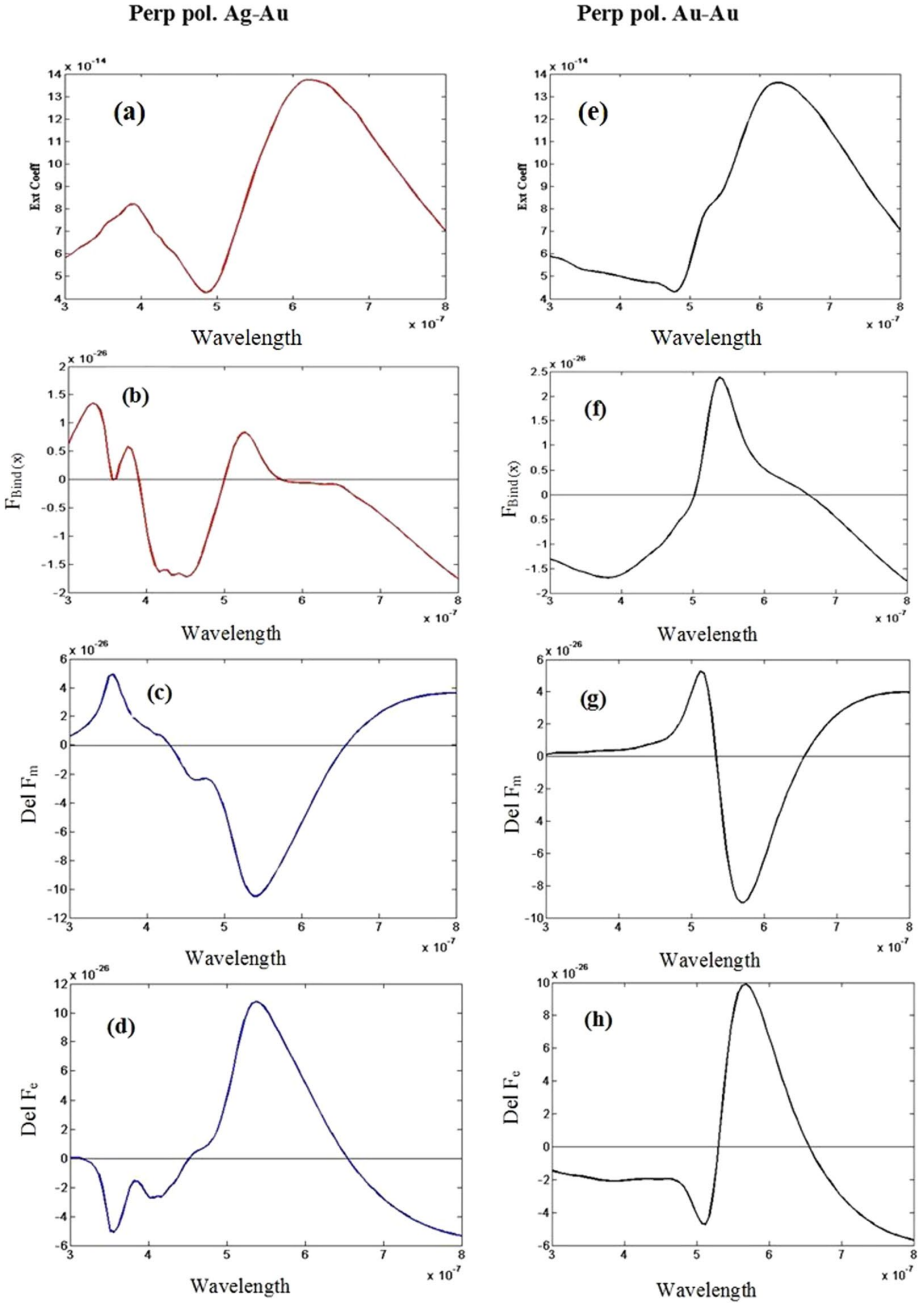
All wavelengths are in meter (m) unit. All Forces are in Newton (N) unit. Considering perpendicular polarized light for the configuration of Fig. 1(a) and ‘*ȹ*’ = 0 degree [on axis Ag-Au]: (**a**) Extinction co-efficient. (**b**) The lateral binding force F_Bind(x)_ = (F_B(x)_−F_S(x)_). (**c**) Difference of bulk Lorentz force. (**d**) Difference of surface force. Considering same polarization of light for the configuration of Fig. 1(b) and ‘*ȹ*’ = 0 degree [on axis Au-Au]: (**e**) Extinction co-efficient (**f**) The lateral binding force F_Bind(x)_ = (F_B(x)_ − F_S(x)_) (**g**) Difference of Lorentz bulk force. (**h**) Difference of Lorentz surface force.
2. It is also demonstrated that whenever the 2^nd^ reversal (positive to negative) of the lateral binding force occurs near the wavelength 500 nm [cf. Fig. 3(a,b) for Ag-Au and Fig. 3(e,f)], the real part of the induced electric dipole moment reverses its sign near the resonance of the smaller object in Supplement S2(b) [which does not occur for parallel polarized case]. So, the reversal of lateral binding force near this specific wavelength can better be explained based on the idea of induced same or opposite electric charges similar to the idea (reversal of the electric polarizability near resonance) proposed in ref.^20^. Results of on-axis Ag-Ag heterodimers are like Ag-Au and Au-Au cases; which have been shown in supplement S3.

### Longitudinal binding force for Off-Axis Heterodimers: end-fire and near end-fire configuration

In this section, we mainly focus on the Ag-Au heterodimers to explain the behaviour of the end fire and near end fire heterodimers [all other cases are shortly listed in Table 1]. Reversal of the optical near field binding force has been observed for only Ag-Au and Au-Au off-axis heterodimers (only when the smaller object perturbs the propagating light at first) and this issue relates to the presence of constructive interference of dipole-quadrupole mode. Another notable point is that: mutual attraction and repulsion of all the off-axis heterodimers can be easily controlled by changing the direction of propagating light or by changing the relative orientation of the particles. All the conclusions of the forthcoming sections have been noted very shortly in Table 1.

### Au-Ag off-axis heterodimers: A general discussion on long. binding force for both polarizations

Now, we consider Au-Ag particles of 100 and 50 nm respectively with inter particle distance of 20 nm [cf. Fig. 1(a) and (d)] but considering that the rotation angle, *φ*, of the particle is between 70 to 110 degrees [i.e. end fire or nearly end fire configuration^20^]. The source is same. We start to create angular displacement from the *x*- axis considering two cases: (A) Rotating the smaller object and fixing the bigger one [cf. Fig. 1(a)] and (B) Rotating the bigger object while keeping the smaller one fixed [cf. Fig. 1(d)]. Now the question arises: ‘Should there be any difference on longitudinal optical binding force for these two cases- (A) and (B)? The notable observation of this work: the behavior of longitudinal binding forces is quite different for these two cases due to the forced breaking of symmetry (due to placing the light source at one side of the dimer configuration instead of at the top of the set-up). If the light source were placed at the top of the set-up, such difference should not arise. According to our forthcoming observations, forced symmetry breaking is detected as one of the key ways to control the inter-particle attraction and repulsion. Some previous symmetry broken set-ups have been discussed in^52,53^ (but not for optical force), which are different than our case.

**Figure 4:**
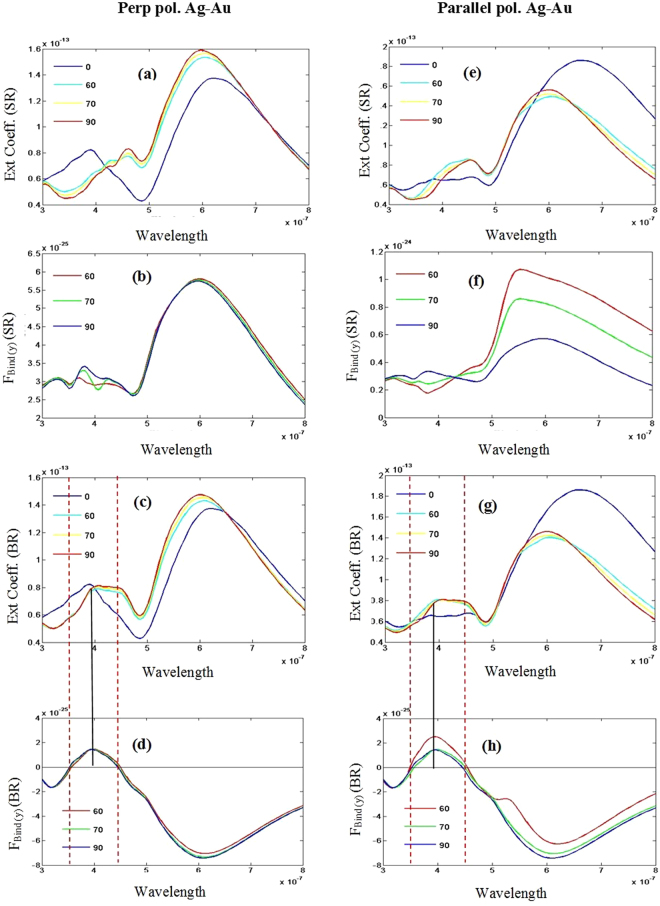
SR and BR represent ‘small rotate’ and ‘big rotate’ respectively and ‘*ȹ*’ = 60, 70 and 90 degree [off axis Ag-Au]. All wavelengths are in meter (m) unit. All Forces are in Newton (N) unit. Considering perpendicular polarized light- for the configuration of Fig. 1(a) (**a**) Extinction co-efficient (SR) (**b**) The longitudinal binding force F_Bind(y)_ (SR); and for the configuration of Fig. 1(d) (**c**) Extinction co-efficient (BR) (**d**) the longitudinal binding force F_Bind(y)_ (BR). Considering parallel polarized light: for the configuration of Fig. 1(a) (**e**) Extinction co-efficient (SR) (**f**) The longitudinal binding force F_Bind(y)_ (SR); and for the configuration of Fig. 1(d) (**g**) Extinction co-efficient (BR) (**h**) The longitudinal binding force F_Bind(y)_ (BR).

However, for both cases- (A) and (B), the extinction cross sections reveal that bonding mode resonance blue shifts for increasing angular displacement in Fig. 4(a,c,e,g) for both of the aforementioned cases [also cf. supplement S4 for the case of Au-Au heterodimers with parallel polarization of light; rotating the smaller object]. It appears that an ‘angular ruler’ may also be possible like previously defined ‘inter-particle gap ruler’ in ref.^54^.

*For the off-axis heterodimers, the attractive force can be defined as the positive value of the optical binding force F*_*Bind*(*y*)_ (*SR*) = (*F*_*S*(*y*)_ − *F*_*B*(*y*)_) *and F*_*Bind*(*y*)_ (*BR*) = (*F*_*B*(*y*)_ − *F*_*S*(*y*)_) *[here SR means small rotating and BR means big rotating]*, considering two important facts: (a) the angular displacement angles should be much higher and *φ* should be as close as 90 degree [i.e. 70 < *φ* < 110] and (b) *x*-directed lateral force F_(x)_ is at least ten times smaller than *y*-directed force F_(y)_ (which is usually satisfied, as the *y*-directed scattering force is usually much higher than the *x*-directed lateral force for plasmonic spherical heterodimers). It should also be noted that the scattering force of the bigger object is always pushing force [negative value of F_B (y)_], which is one of the key issues to control the reversal of the *y*-directed binding force (this will be explained next).

#### Au-Ag off-axis heterodimers: Rotating the smaller particle and fixing the big one

At first, we consider the rotation of the smaller object [case (A); cf. Fig. 1(a)] for both perpendicular and parallel polarizations of the light. For *φ* = 60 to 90 degrees, it is observed that only the scattering force of the smaller object experiences the reversal at bonding resonance region. On the other hand, scattering force of the bigger object (F_B(y)_) is always pushing force. But the most important fact is that $|{F}_{S(y)}| < |{F}_{B(y)}|$; always. As a result, F_Bind(y)_ (SR) = (F_S(y)_ − F_B(y)_) is always *positive* [*attractive force* (defined previously) as shown in Fig. 4(b and f)]. Importantly, the real part of electric dipole moment of the smaller object reverses its sign near the bonding resonance [not shown] but F_Bind(y)_ (SR) always remains attractive with no reversal of sign. In fact, the difference of the particle radius of both the particles plays a vital role. When one of the particles in the heterodimer is much larger than the other one and the propagating light is perturbed by the bigger object at first, the scattered field from the larger particle enhances much^20^ and this field enhancement is quite high at the inter particle gap position. This enhanced field forces the surface plasmon polariton to confine in the surface of the big particle and decay exponentially outside it. Ultimately this forms an intensity gradient field near the particle. Thus, when the second particle (smaller one) comes into the gradient field (in end-fire and nearly end-fire set-up), it experiences the usual intensity gradient force. i.e., the binding force between them is always attractive in this case.

#### Au-Ag off-axis heterodimers: Rotating the bigger particle keeping the small one fixed

Now, we consider the alternate orientation [case (B); cf. Fig. 1(d)] by rotating the bigger object and fixing the smaller object. For this configuration, F_Bind(y)_ (BR) reverses during the antibonding type resonance mode and near spectral dip position. This is explained next.

**Figure 5:**
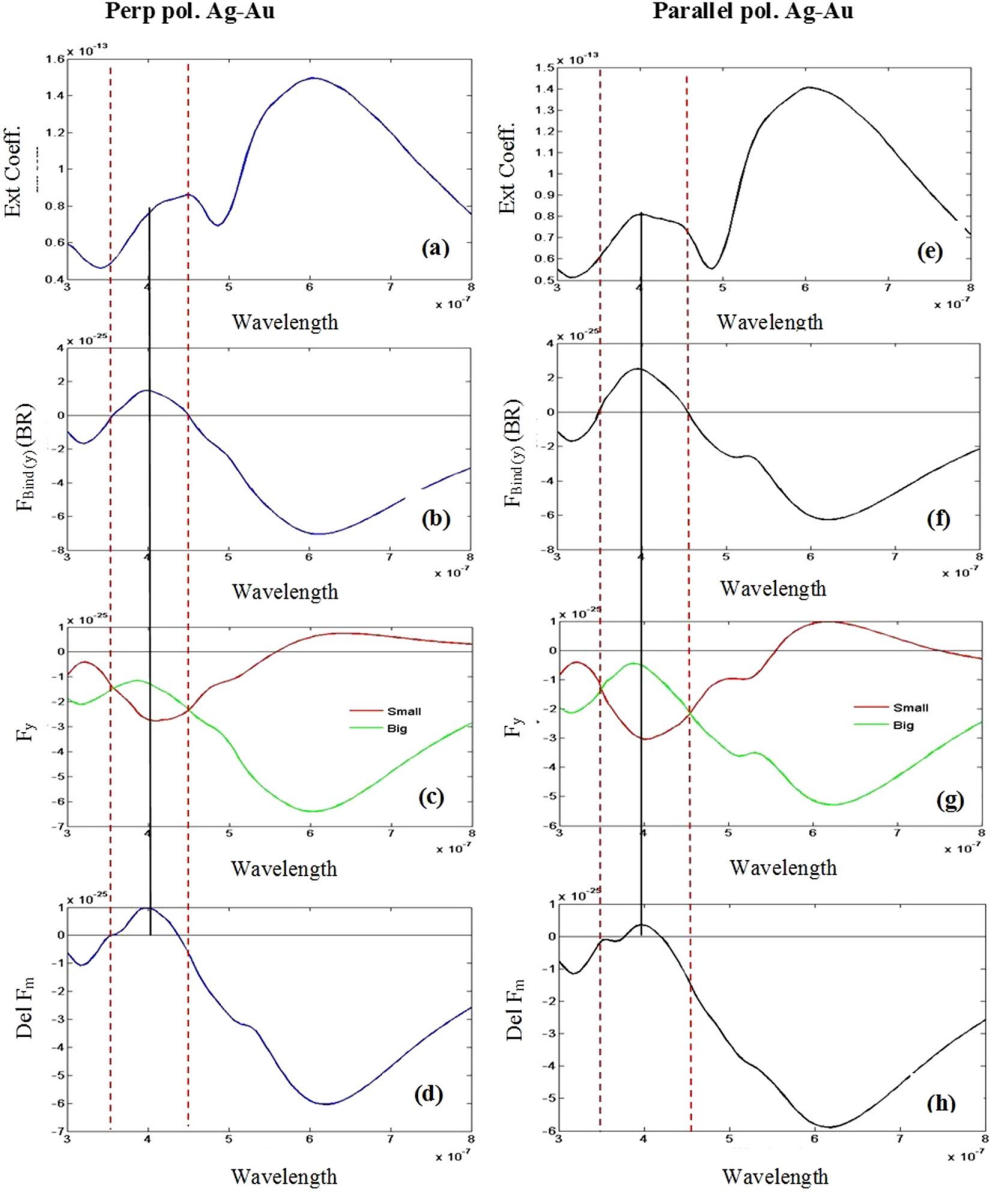
SR and BR represent ‘small rotate’ and ‘big rotate’ respectively. All wavelengths are in meter (m) unit. All Forces are in Newton (N) unit. For off axis Ag-Au and BR [the configuration of Fig. 1(d) and ‘*ȹ*’ = 70 degree]: Considering perpendicular polarized light (**a**) Extinction co-efficient (**b**) The longitudinal binding force F_Bind(y)_ (BR) (**c**) Time averaged force on each particle. (**d**) Difference of bulk Lorentz force. Considering parallel polarized light for same configuration (**e**) Extinction co-efficient (**f**) The longitudinal binding force F_Bind(y)_ (BR) (**g**) Time averaged force on each particle. (**h**) Difference of bulk Lorentz force.

When the smaller object is rotated and the propagating light is perturbed by the bigger object at first, the scattering force on the bigger object (always pushing) is always higher than the smaller one. But when the bigger object is rotating and the propagating light is perturbed by the smaller object at first, there are some chances to find some wavelength regions when the scattering force on the smaller object becomes higher than the bigger object. In this way, the binding force F_Bind(y)_ (BR) = (F_B(y)_ − F_S(y)_) can be observed *attractive* [*positive value as defined previously*] in those wavelength regions. This is what exactly happens during the anti-bonding type resonance modes as shown in Fig. 4(c,d and g,h); which is quite different than the conventional idea of optical binding force with homodimers^24^. For homodimers, according to the quasi-static approximation limit^24^: the bonding modes and antibonding modes have positive and negative definite slopes, respectively. As a result, consequently they must, respectively, induce attraction and repulsion. But we clearly observe the opposite scenario for the heterodimer set (at a fixed edge to edge distance of 20 nm) when the light is perturbed by the smaller object at first. Then the question arises why this kind of opposite behavior is observed for such ‘forced’ symmetry broken heterodimer set-ups. Its answer lies in the electrodynamics calculations and force distribution analysis instead of the quasi-static analysis; mainly due to the generation of multipoles. Based on the results demonstrated in Fig. 5(a–h) we shall discuss the detail dynamics considering a specific case: *φ* = 70 degree.

**Figure 6:**
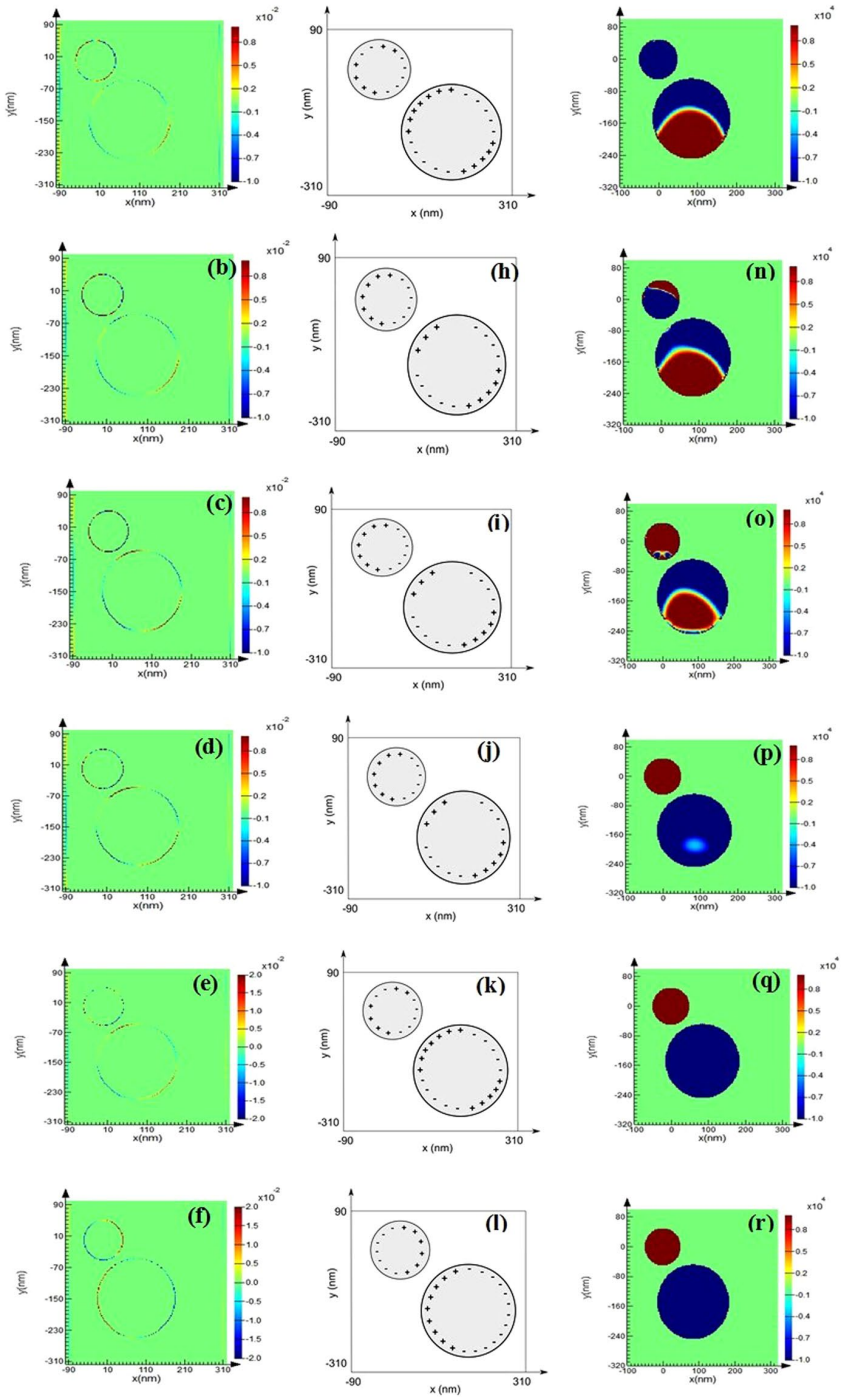
For off axis Ag-Au and by rotating the big particle [the configuration of Fig. 1(d) and ‘*ȹ*’ = 70 degree]: Considering parallel polarized light, from left first two columns represent surface charges [(**a**–**l**)] and the third column represents steady state current [(**m**–**r**)]. We have chosen six wavelengths for six different rows (from top to bottom): 338, 354, 400, 457, 485 and 612 nm. Charge distributions: (**a**) QQ (**b**) DQ (**c**) DQ (**d**) DQ (**e**) QQ (**f**) DD; where Q and D mean quadrupole and dipole respectively.

In Fig. 5(d) and (h) we have plotted the difference of the bulk Lorentz force, which clearly suggests that the total binding force is dominated by the bulk part of Lorentz force [which is in contrast with the commonly observed dominance of surface^34^/polarization charge induced force^35^ for plasmonic objects]. This force can be considered as the scattering force part^34,55^ of the total force, which is physically originating from the multiple scattering between the smaller and the bigger object. Figure 5(c) and (g) suggest that during the anti-bonding resonance mode, the directive forward scattering of the bigger object is much higher than the smaller object. Surface charge plots in Fig. 6 suggest that for the parallel polarized illumination, during the wavelength spectrum around 350 nm to 470 nm, constructive interference occurs due to dipole quadrupole resonance. Though this is not super-scattering^3^, it is recognized that the forward scattering of the bigger object increases during these spectra [cf. the extinction spectra in Fig. 4(g) where the magnitude of extinction co-efficient increases for higher rotation angles during this specific spectrum regime]. On the other hand, exactly opposite scenario takes place for the bonding mode resonance. For example, at higher wavelength regime during bonding mode resonance the smaller object even experiences optical pulling force [cf. Fig. 5(c) and (g)] because of: (i) very strong effective forward scattering along with (ii) more reflected light from the bigger object.

It is observed that the reversal wavelength of the optical binding type force F_Bind(y)_ (BR) = (F_B(y)_ − F_S(y)_) remains almost fixed along with the spectral dip position [cf. Fig. 4(c,d and g,h)], though the bonding mode resonance blue shifts gradually with the rotation of the bigger object. Moreover, very similar to ref.^27^ the reversal of the phase of the steady state current takes place near the spectral dip in our heterodimer set-ups as shown in Fig. 6(m–r), though it is constructive dipole quadrupole resonance instead of destructive one reported in^27^.

### Final remarks: Simplest procedure to reverse the long. binding force for the heterodimers

So far, we have observed that:

- *When the bigger object is rotated and the propagating light is perturbed by the smaller object at first*, for only Ag-Au and Ag-Ag off-axis heterodimers reversal of longitudinal binding force occurs [i.e. the dynamics of Ag-Ag and Au-Au heterodimers have been discussed in detail in Supplement S5]. Especially after the anti-bonding resonance mode, the longitudinal binding force is observed always *repulsive* for such heterodimers. On the other hand, for Au-Au heterodimers this force is always *repulsive* for such configuration for the visible wavelength spectrum.
- In contrast, *when the smaller object is rotated and the propagating light is perturbed by the bigger object at first*, for all the spherical heterodimers no reversal of longitudinal binding force occurs. Binding force is always *attractive* for such configuration.

Based on the aforementioned two simple observations of (a) and (b), finally we can conclude *for the higher wavelength regions* (*after anti-bonding resonance*): (1) if we change the direction of propagating light manually by bringing the light source from one side of the dimers to another side, it will be easily possible to observe the mutual repulsion [for the configuration of Fig. 2(a)] and attraction [for the configuration of Fig. 2(b)] of all the heterodimer sets just due to the automatic change of the relative dimer position of smaller and bigger objects. Or (2) simply by changing the relative orientation of the heterodimers manually (not light propagation direction), it is also possible to observe such reversal [cf. Fig. 2(a) and (c)]. As per we understand, such simplest control of near field binding force reversal may be impossible with the plasmonic homo-dimers^22–26^.

Though the reversal [i.e. the controlled attraction and repulsion of the particles] of near field longitudinal binding force depend on the size of each plasmonic particle and also the inter-particle gap between them (It is important to note that we are considering ‘near field’ binding force); such dependencies should be much relaxed for our set-ups in comparison with previously reported situations^20^ according to our proposed aforementioned final two techniques.

## Conclusions

In this work, (a) at first we have demonstrated that Fano resonance^1,4^ does not contribute to binding force reversal for spherical plasmonic heterodimers. As a result, we conclude that Fano resonance may not be considered as a universal process of the reversal of near field binding force. (b) Later, it is also demonstrated that even with the presence of both bonding and^2^ anti-bonding modes^24^, reversal of binding force may not occur for spherical plasmonic heterodimers.

As both (a) and (b) do not favor the reversal of near field binding force, we have introduced the idea of ‘forced breaking of symmetry’ for heterodimers and later achieved the reversal of binding force for our different set-ups. We have listed some cases of such reversal in Table 1 based on the observation: *the reversal of lateral and longitudinal binding force follow fully different mechanisms*. However, Table 1 represents only few possible cases which ultimately lead to the possible final/main conclusion of this article: ‘the reversal of near field longitudinal binding force can be easily controlled (*at higher wavelength regions after anti bonding resonance*) due to forced symmetry breaking: (i) just by changing the direction of light propagation for a specific set-up of off-axis heterodimers or (ii) by changing their relative orientation.’ Remarkably, this final proposal may also open a novel way to investigate: whether such controlled reversal of near field longitudinal optical binding force is possible for other shaped heterodimers or not.

Although plasmonic symmetry breaking, optical trapping based on symmetry breaking, plasmonic focusing and Fano resonance have been investigated in several works^52,53,56–58^, the possible control of optical binding force based on the forced symmetry breaking is still not available in literature. In addition, the demonstrations throughout our article are quite different than the previous observations of binding force reversal of the homodimers reported in^22–26^. Our proposals in this article, controlling the attraction and repulsion of near field binding force, can be very useful for improved sensors^4–6^, particle clustering and aggregation^22–24^.

## Electronic supplementary material

Supplementary Information
