# Supplementary material for: Plasmonic Spherical Heterodimers: Reversal of Optical Binding Force Based on the Forced Breaking of Symmetry
Source: Sci Rep. 2018 Feb 16;8:3164. doi: 10.1038/s41598-018-21498-4 (PMC5816674; doi:10.1038/s41598-018-21498-4)
Supplement: Supplementary file 1 — Supplementary Information [file 41598_2018_21498_MOESM1_ESM.pdf]

Supplement Information of "Plasmonic Spherical Heterodimers: Reversal of Optical Binding Force Based on the Forced Breaking of Symmetry"

M.R.C. Mahdy*[1,2,3], Md. Danesh[2], Tianhang Zhang[2,4], Weiqiang Ding*[5], Hamim Mahmud Rivy[1], Ariful Bari Chowdhury[6], MQ Mehmood[7,2]

[1]*Department of Electrical & Computer Engineering, North South University, Bashundhara, Dhaka 1229, Bangladesh*

[2]*Department of Electrical and Computer Engineering, National University of Singapore, 4 Engineering Drive 3, Singapore 117583*

[3]*Pi Labs Bangladesh Ltd., ARA Bhaban, 39, Kazi Nazrul Islam Avenue, Kawran Bazar, Dhaka, Bangladesh*

[4]*NUS Graduate School for Integrative Sciences and Engineering, National University of Singapore, 28 Medical Drive, Singapore 117456*

[5]*Department of Physics, Harbin Institute of Technology, Harbin 150001, People's Republic of China*

[6]*Department of Public Health, North South University, Bashundhara, Dhaka 1229, Bangladesh*

[7]*Department of Electrical Engineering, Information Technology University of the Punjab, 54000 Lahore, Pakistan*

[*] Corresponding author: mahdy.chowdhury@northsouth.edu and wqding@hit.edu.cn

S1. On-axis and off-axis configurations of heterodimers:

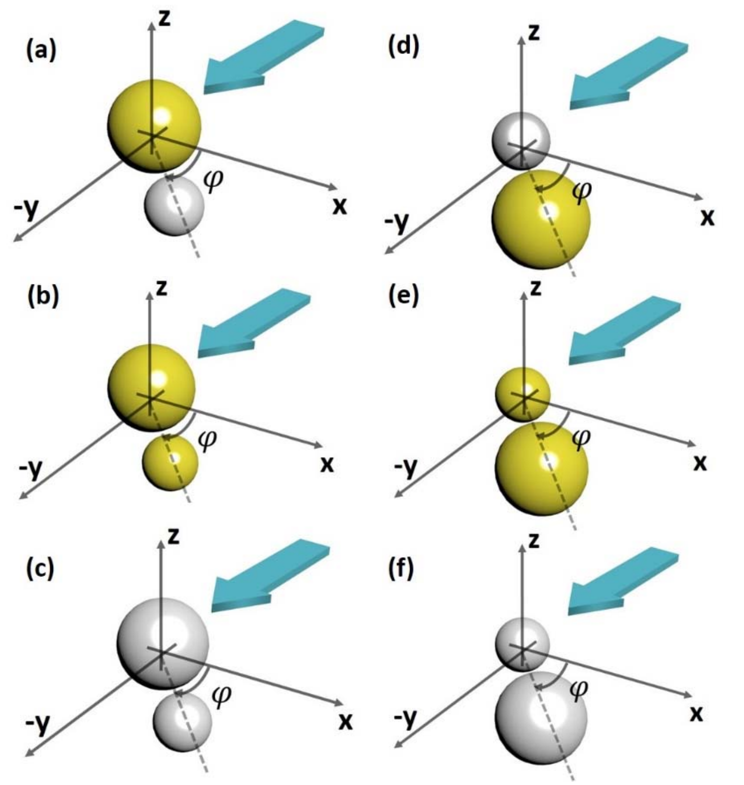

Fig.1s: Detail caption is given in Fig.1 in the main article where: (a) Au-Ag (b) Au-Au (c) Ag-Ag (d) Ag-Au (e) Au-Au and (f) Ag-Ag.

S2. Lateral optical force on Ag-Au and Au-Au heterodimers for on-axis configuration

(a) Parallel Polarization: No reversal of lateral binding force for Au-Ag and Au-Au on-axis heterodimers

At first, we consider two on-axis [$\varphi = 0$] Ag-Au and Au-Au hetero-dimer set-ups of 100 nm and 50 nm with inter particle distance of 20 nm [cf. Fig. 1s (a), (b) in the supplement] and parallel polarized light. Lateral binding force reversal does not occur for the light polarized parallel to the dimer axis [cf. Fig. 2s in the supplement]. Some remarkable facts are:

When Au-Au heterodimers of 76 nm and 50 nm are placed at 4nm distance and are shined with parallel polarization, Fano resonance takes place (cf. the case-2 in [1]). Similarly, for Ag-Au heterodimers such Fano resonance has been demonstrated in [2]. We have observed that even for such cases no reversal of the optical binding force occurs when the light polarization is parallel to dimer axis.

Moreover, a plasmonic "heterodimer" structure is expected to support both bonding and antibonding plasmon modes at the same time for both the transverse and longitudinal polarization due to its broken symmetry [3]. It is notable that for the parallel polarization, both the bonding and anti-bonding mode arises for the hetero-dimers [3]; but we have observed no reversal of optical binding force for Ag-Au or Au-Au or Ag-Ag hetero-dimers. As a result, in general, the reversal of lateral optical binding force

for hetero-dimers (which will be shown in the next sub section) cannot be explained based on the idea of bonding and anti-bonding mode [4], too.

So, the important conclusion is that although reversal of optical binding force occurs for nano rods or other shapes due to Fano resonance [5, 6], Fano resonance is in general not the reason of the reversal of optical binding force. So, particle size/shape, material and light polarizations are indeed important factors for such binding force reversal.

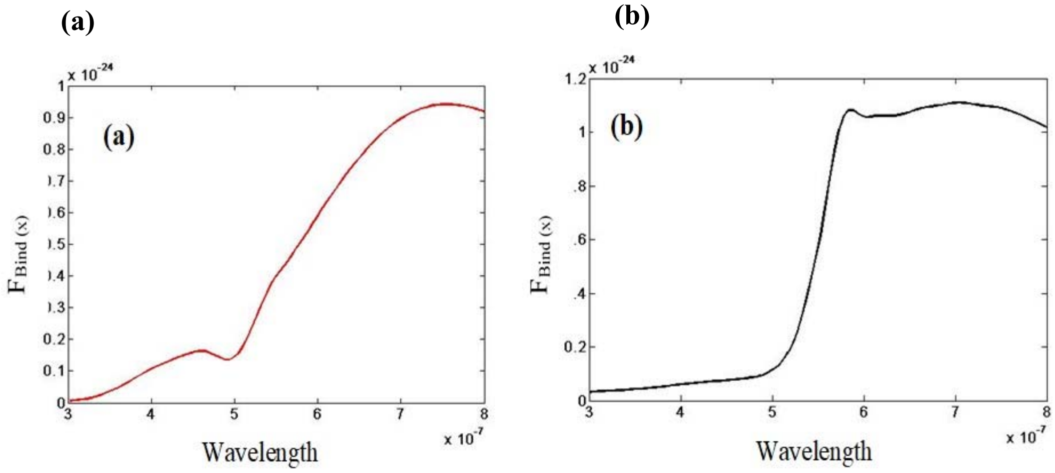

Fig. 2s: Considering parallel polarized light the lateral binding force $F_{Bind\ (x)} = (F_{B\ (x)}\text{-}F_{S\ (x)})$ and 'φ' = 0 degree: (a) for the configuration of Fig.1s (a) [on axis Ag-Au] (b) and for the configuration of Fig.1s (b) [on axis Au-Au]. All wavelengths are in meter (m) unit. All Forces are in Newton (N) unit.

(b) Perpendicular Polarization: Reversal of lateral binding force for Au-Ag and Au-Au on-axis heterodimers

Again, we consider two on-axis Ag-Au and Au-Au particles of 100 ad 50 nm with inter particle distance of 20 nm [cf. Fig. 1s (a), (b))] and perpendicular polarized light. The extinction cross section in Fig. 4(a) and (e) in the main article clearly reveal that both bonding resonance and anti-bonding resonance are occurring [3]. The mechanism of the force reversal of such hetero-dimers will be explained here shortly.

(1) At higher wavelength region (near 650 nm wavelength): it is observed that near the bonding resonance mode, reversal of the optical binding force, $F_{Bind\ (x)} = (F_{B\ (x)}\text{-}F_{S\ (x)})$ occurs at the wavelength 646 nm. Here $F_{B(x)}$ and $F_{S(x)}$ are the +*x* directed time averaged force on big and small particle respectively. At this specific wavelength two different hetero-dimers do not oscillate at lateral direction and the effective optical molecule does not experience any surface and bulk Lorentz force. The difference of the scattering part [cf. Eq (4) in the main article] or bulk part of the total Lorentz force [7,8] on a plasmonic object should describe the relative bulk force experienced by the optical molecule [Eq (5) given in main article]:

$$\text{Del } F_{\text{Bulk}(x)} = \int [\langle \boldsymbol{f}_{\text{Bulk}\,(B)} \rangle dv_{(B)}] - \int [\langle \boldsymbol{f}_{\text{Bulk}\,(S)} \rangle dv_{(S)}] \qquad (1\text{s})$$

Here; subscript (x), (B) and (S) represent: +x direction, bigger object and smaller object respectively. At the same time the difference of the gradient part [9] [which originates from induced surface charges; cf. Eq (3) in main article] of the total Lorentz force [7,8] on a plasmonic object should describe the relative surface force experienced by the optical molecule [Eq (6) given in main article]:

$$\text{Del F}_{\text{Surf (x)}} = \int [\langle \boldsymbol{f}_{\text{Surface}\,(B)} \rangle ds_{(B)}] - \int [\langle \boldsymbol{f}_{\text{Surface}\,(S)} \rangle ds_{(S)}] \tag{2s}$$

At wavelength 646 nm, the right-hand sides of both Eqs (1s) and (2s) in this supplement are approximately zero. It should be noted that: $F_{Bind\ (x)} = \left(F_{B\ (x)} - F_{S\ (x)}\right) = \text{Del F}_{\text{Bulk}(x)} + \text{Del F}_{\text{Surf}\,(x)}$. It is also observed that the phase of the steady current and surface charges rapidly change just before and after this specific wavelength 646 nm as shown in Fig. 3s.

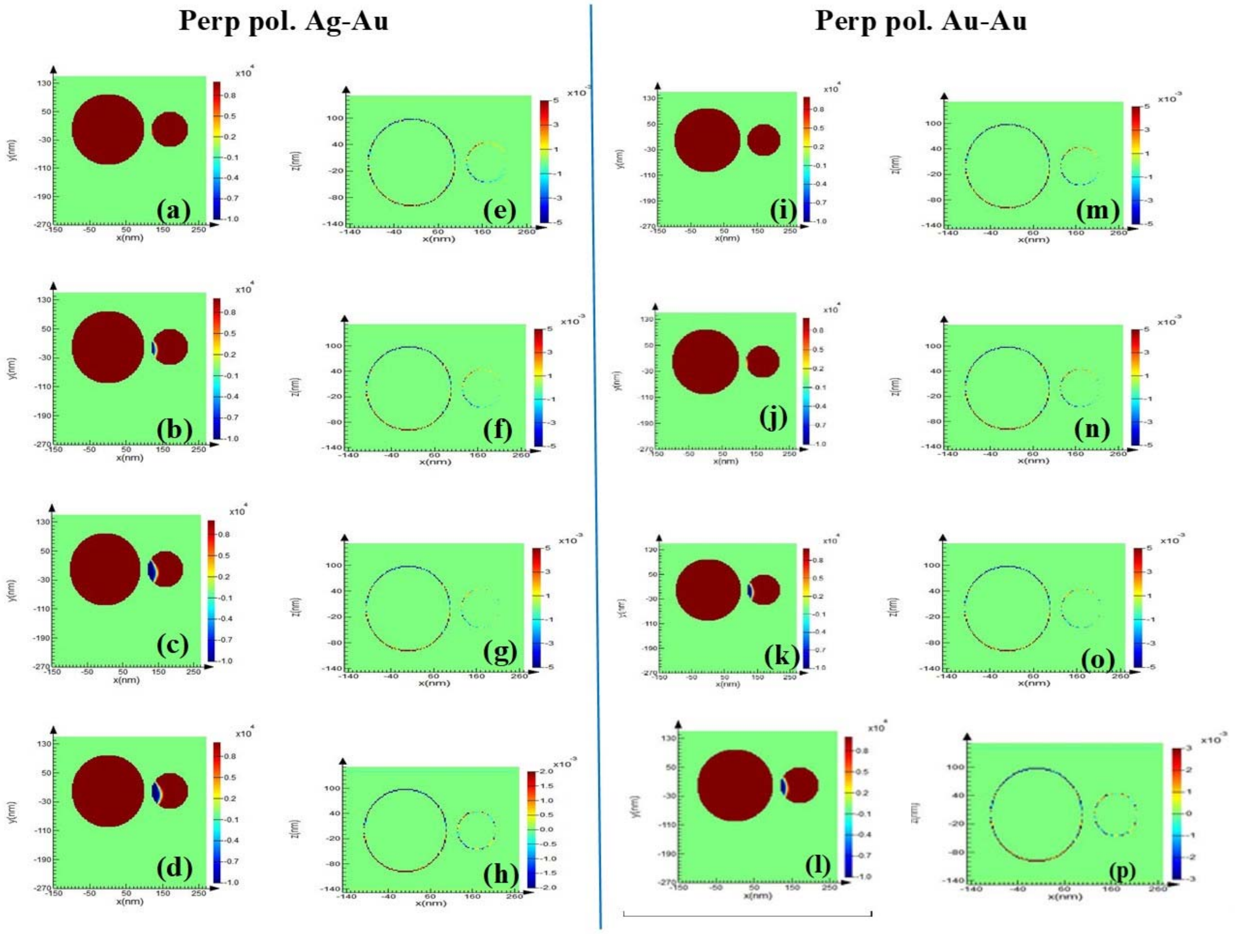

Fig.3s: Steady state current (J_z) and charge distribution have been illustrated for on-axis Ag-Au and Au-Au (perpendicular polarized light) for four different wavelengths- 628 nm: 1st row (from top); 646 nm: 2nd row; 664 nm: 3rd row; 684 nm: 4th row . Del $F_{Surf\ (x)}$ and Del $F_{Bulk\ (x)}$ become nearly zero at 646 nm and the lateral binding force reversal starts to occur form that wavelength which is very close to the bonding resonance wavelength. Before and after 646 nm, the steady current and surface charges are different in two different particles; especially for surface charges: In the lower wavelength than 646 nm and at the wavelength 646 nm resonance is DD type but slowly turns into DQ type at 684 nm [D and Q represent dipole and quadrupole respectively]. Interestingly quadrupole arises in the smaller object at wavelength around 684 nm.

In short, based on the observations we can explain the reversal of lateral binding force in higher wavelength region (after the dipole-dipole resonance region) in this way: the shorter object's dipolar resonance behavior turns into quadrupole resonance behavior at much higher wavelength region [usually this is not common for shorter sized objects] and this continuous process [from DD resonance to DQ resonance] ultimately affects both the behavior of surface charges [bound and free] and bulk currents [bound and free] of the objects. For example- a sudden change in the phase of current is observed along with the change of the surface charges for this heterodimer set (especially for the smaller object). The surface charge is connected with surface force part of Lorentz force and the bulk current with the bulk Lorentz force. Both of the forces (their individual difference $\text{Del F}_{\text{Bulk}(x)}$ and $\text{Del F}_{\text{Surf}(x)}$) face a rapid change of sign as shown in several figures in main article [Fig. 3 ©, (d) and (g), (h)]. This clearly indicates that the reversal of the 'overall' binding force is arising mostly due to the behavioral change of surface charges and currents of the plasmonic objects, which is connected with the transformation of the induced resonance process [i.e. from DD to DQ].

(2) At lower wavelength region (near 500 nm wavelength): for the transverse/perpendicular polarization case, in our Ag-Au, Au-Au and Ag-Ag heterodimer set-up, though spectral dip is observed at certain wavelengths, bonding dipole quadrupole (BDQ) pattern at the spectral minimum regions [1] is not found for the charge distribution of the objects (also cf. the discussion in [2] for perpendicular polarized light). As a result, this spectral minimum cannot be identified as Fano resonance/ Fano dip. We now consider a different idea [10]: the electric dipole moment of the objects to explain the reversal of binding force based on same and opposite electric charges. This idea of electric dipole moment [10] should be a more generalized idea than the electric polarizability (discussed in ref. [11]), as the overall size of the dimer set-up is higher than the dipolar limit in this work. The real part of electric dipole moment of an object is defined as [10]:

$$p = \mathrm{Re}\int [i\omega(\varepsilon_s - \varepsilon_0)\boldsymbol{E}_{\mathrm{in}}]dv \tag{3s}$$

It is observed that whenever the reversal of the lateral binding force occurs for transverse/perpendicular polarization, the real part of the electric dipole moment reverses its sign near the resonance for both the smaller object as shown in Fig 4s (a) and (c). On the other hand, it is observed that whenever no reversal of the lateral binding force occurs for longitudinal/parallel polarization, the real part of the electric dipole moment does not reverse their sign near the resonance for the smaller objects as shown in Fig 4s (b) and (d). So, the reversal of lateral binding force near this specific wavelength [near 500 nm as shown in Fig 4(b) and 4(f) in the main article] can better be explained based on the idea of induced same or opposite electric charges due to induced electric resonance [10] similar to the idea (reversal of the electric polarizability near resonance) proposed in ref. [11].

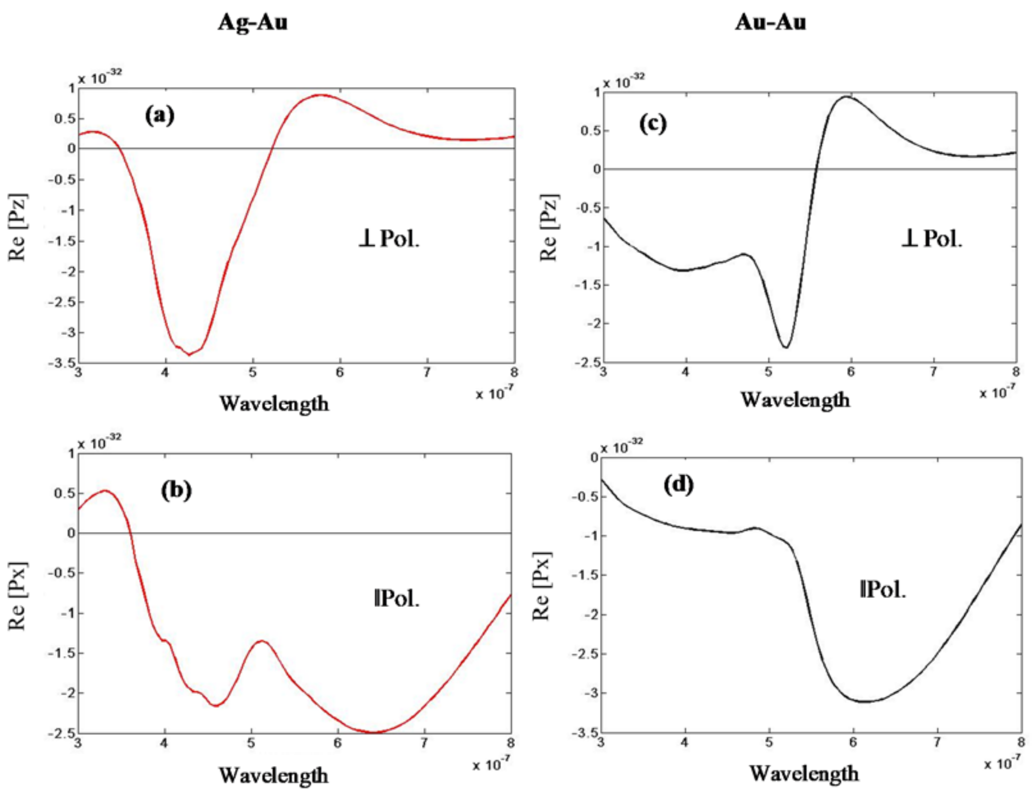

Fig. 4s: For both polarizations, electric dipole moment of Ag-Au heterodimers [for configuration of Fig. 1s (a)] and Au-Au heterodimers [for config. of Fig. 1s (b)]. All wavelengths are in meter (m) unit.

S3. Lateral Binding Force of on-axis Ag-Ag heterodimers:

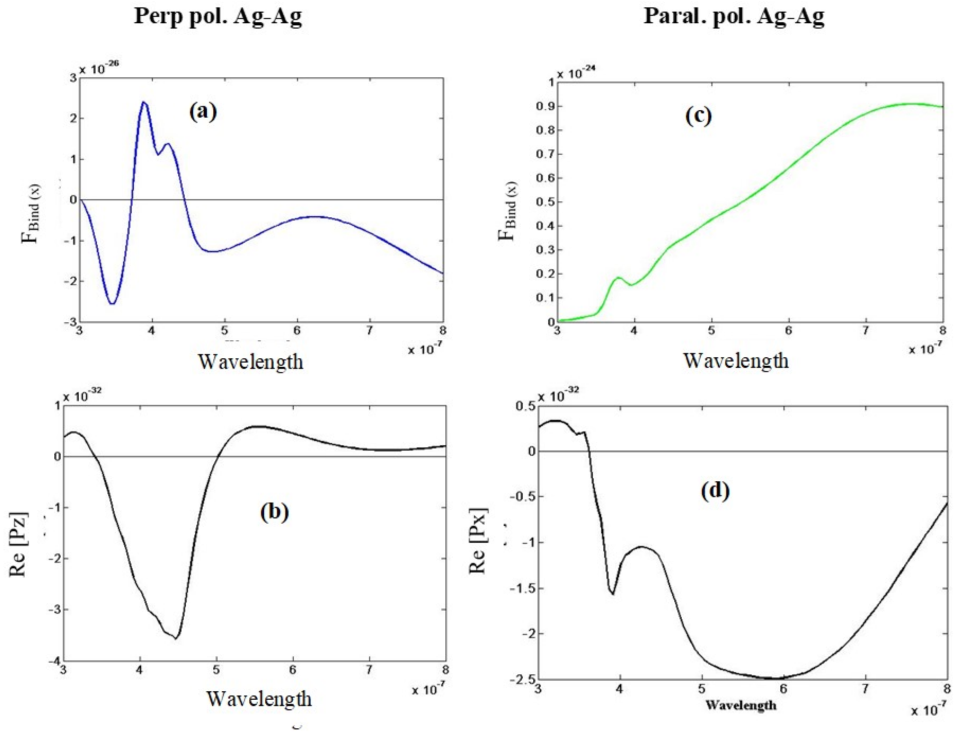

Fig. 5s: (a) and (c): Lateral binding force for on-axis Ag-Ag heterodimers [cf. Fig. 1s (c)]. (b) and (d): electric dipole moment of on-axis Ag-Ag heterodimers. All wavelengths are in meter (m) unit.

S4. Blue shift of bonding resonance mode due to the rotation of one particle in set:

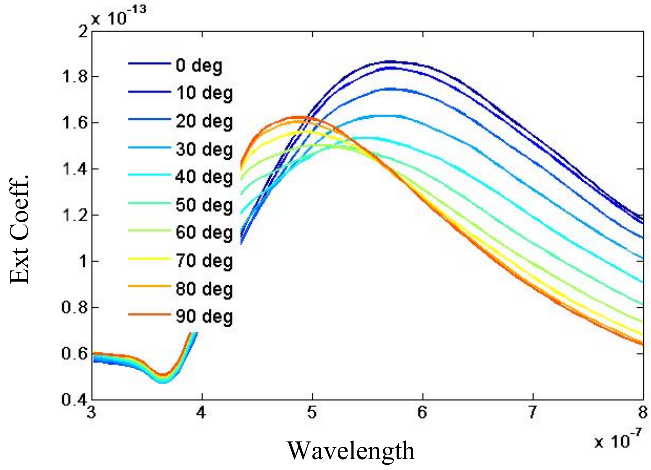

Fig 6s: Blue shift of Bonding Resonance mode due to the rotation [φ =0 to 90 deg] of the bigger particle of Au-Au heterodimer set in Fig. 1s (b) [parallel polarized light]. All wavelengths are in meter (m) unit.

S5. Rotation of Bigger Particle and longitudinal binding force for Ag-Ag and Au-Au off-axis heterodimers:

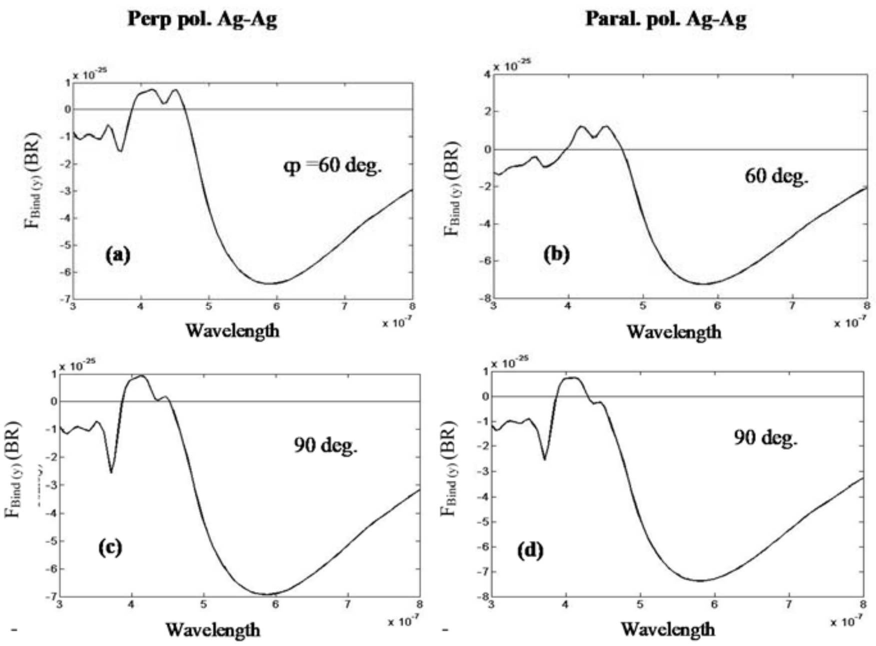

Fig 7s: Reversal of longitudinal binding force for off-axis Ag-Ag heterodimers [cf. Fig. 1s (f)] for both polarizations. All wavelengths are in meter (m) unit. All Forces are in Newton (N) unit.

When the smaller particle is rotated keeping the bigger particle fixed, no reversal of the longitudinal binding force occurs for Ag-Ag and Au-Au off-axis [i.e. 70< φ <110] hetero-dimers similar to Ag-Au hetero-dimers discussed in main article. However, when the bigger object is rotated keeping the smaller one fixed for Au-Au and Ag-Ag hetero-dimers [cf. Fig. 1s (e) and (f) respectively], in Fig. 7s it is shown that for both polarizations of light, Ag-Ag off-axis heterodimers support the binding force reversal similar to Ag-Au hetero-dimers discussed in main article but Au-Au heterodimers do not support such reversal as shown in Fig. 8s [reason: the –y directed pushing force on the bigger object is always much higher than the smaller one and anti-bonding type resonance almost vanishes according to Fig. 8s (a), (c)]. Charge distributions of off-axis Au-Au hetero-dimer set [given in Fig. 9s] is observed slightly different than the Ag-Au off-axis hetero-dimer set discussed in the main article and it is clearly observed that constructive interference due to dipole quadrupole mode does not occur for Au-Au off-axis heterodimers [i.e. cf. the extinction spectra of φ =90 and 0 degree in Fig 6s in Supplement S4 for the case of parallel polarized light; no anti-bonding type mode arises in the extinction spectra].

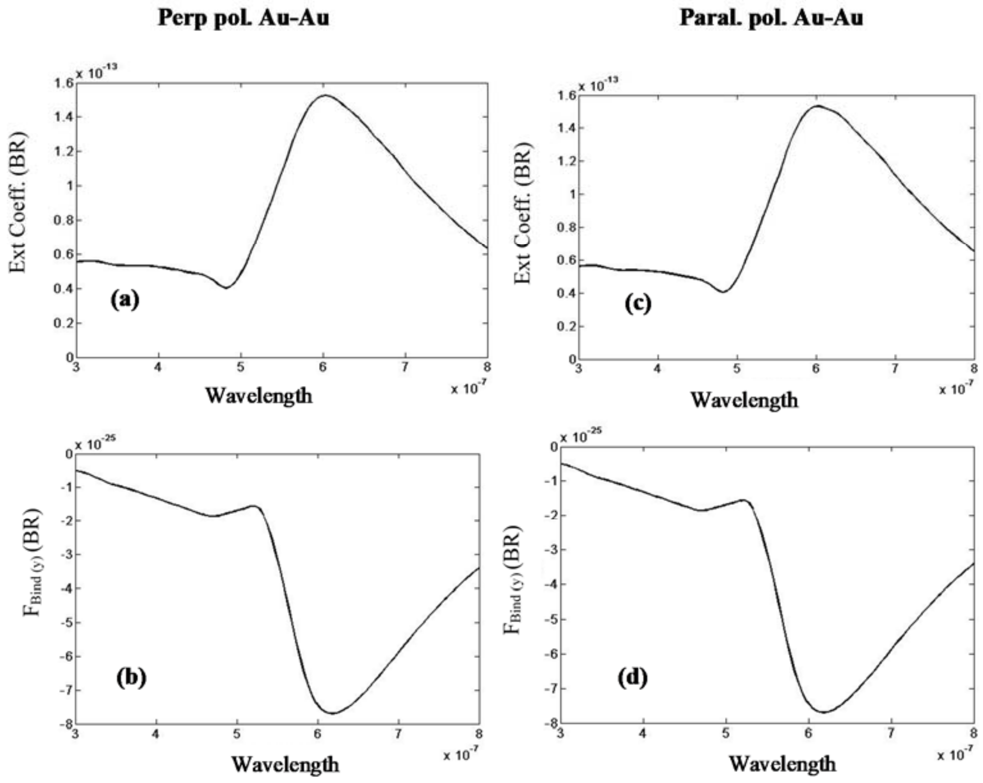

Fig. 8s: (a) and (c): extinction coefficient of off-axis [only φ =90 degree case shown here] Au-Au hetero-dimers [cf. the configuration of Fig. 1s (e)]. (b) and (d): Longitudinal binding force on off-axis [only φ =90 degree case shown here] Au-Au hetero-dimers. All wavelengths are in meter (m) unit. All Forces are in Newton (N) unit.

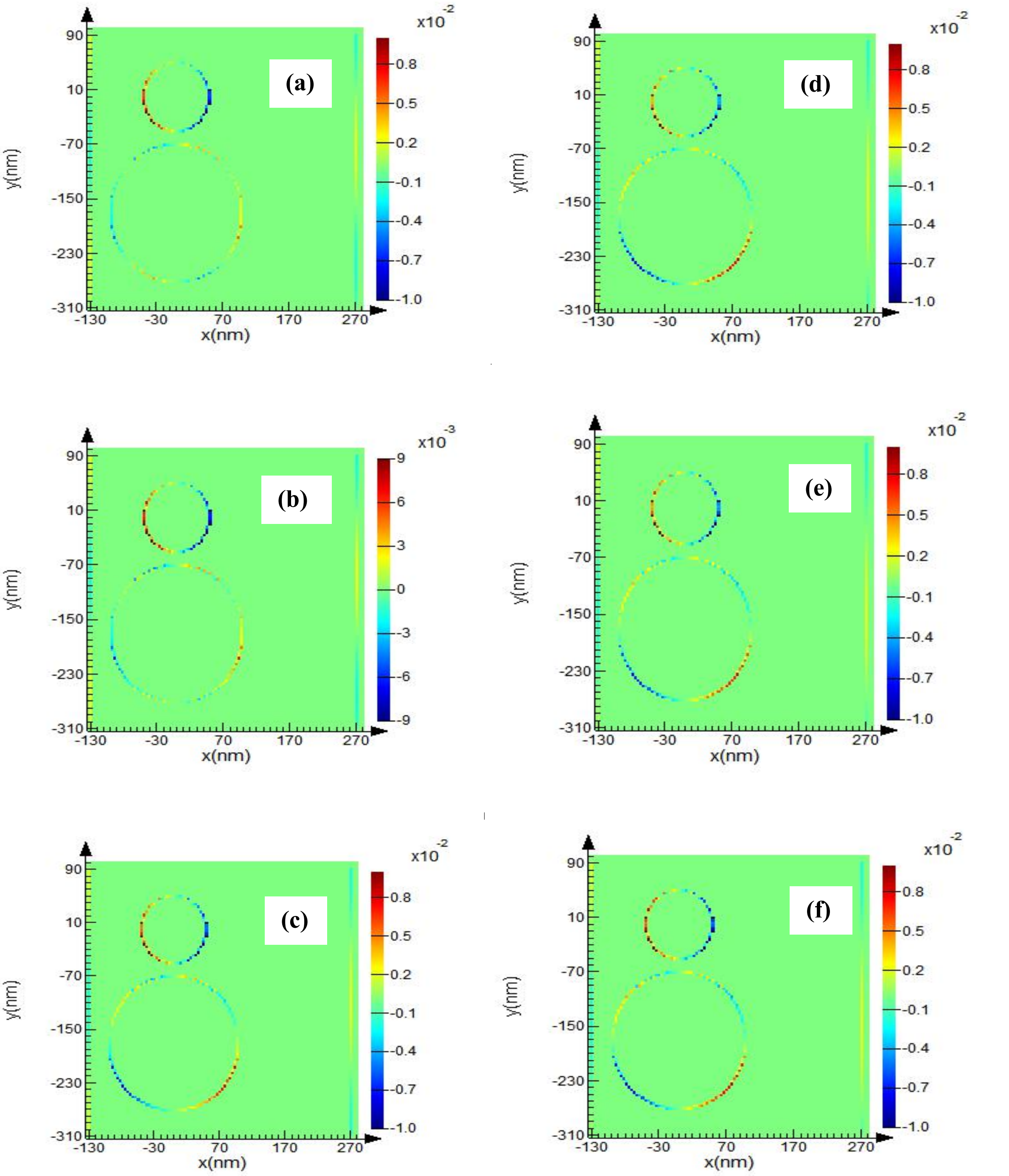

Fig 9s: For off axis Au-Au and by rotating the big particle [the configuration of Fig.1s (e) and ‘φ’ = 90 degree]: Considering parallel polarized light surface charges [(a)-(f)].We have chosen six wavelengths from (a) to (f): 338, 354, 4, 457, 476 and 485 nm. Charge distributions: (a) DD (b) DD (c) DQ (d) DQ (e) DQ (f) DQ; where Q and D mean quadrupole and dipole respectively.

REFERENCES

[1] Brown, L. V., Sobhani, H., Lassiter, J. B., Nordlander, P. & Halas, N. J. Heterodimers: plasmonic properties of mismatched nanoparticle pairs. *Acs Nano* **4**, 819 (2010).

[2] Peña-Rodríguez, O., Pal, U., Campoy-Quiles, M., Rodríguez-Fernández, L., Garriga, M. & Alonso, M. I. Enhanced Fano resonance in asymmetrical Au: Ag heterodimers. *The Journal of Physical Chemistry C* **115**, 6410 (2011).

[3] Sheikholeslami, S., Jun, Y. W., Jain, P. K. & Alivisatos, A. P. Coupling of optical resonances in a compositionally asymmetric plasmonic nanoparticle dimer. *Nano Lett.* **10**, 2655 (2010).

[4] Ng, J., Tang, R. & Chan, C. T. Electrodynamics study of plasmonic bonding and antibonding forces in a bisphere. *Phys. Rev. B* **77**, 195407(2008).

[5] Zhang, Q., Xiao, J. J., Zhang, X. M., Yao, Y. & Liu, H. Reversal of optical binding force by Fano resonance in plasmonic nanorod heterodimer. *Opt. Express*.**21**, 6601(2013).

[6] Zhang, Q. & Xiao, J. J. Multiple reversals of optical binding force in plasmonic disk-ring nanostructures with dipole-multipole Fano resonances. *Optics letters* **38**, 4240 (2013).

[7] Wang, M., Li, H., Gao, D., Gao, L., Xu, J. & Qiu, C. W. Radiation pressure of active dispersive chiral slabs. *Opt. Express*.**23**, 16546 (2015).

[8] Chen, H., Zhang, B., Luo, Y, Kemp, B. A., Zhang, J., Ran, L. & Wu, B. I. Lorentz force and radiation pressure on a spherical cloak. *Phys. Rev. A* **80**, 011808 (2009).

[9] Min, C., Shen, Z., Shen, J., Zhang, Y., Fang, H., Yuan, G., Du, L., Zhu, S., Lei, T. & Yuan, X. Focused plasmonic trapping of metallic particles. *Nat. comm.* **4**, 2891 (2013) and R.Quidant, C.Girard, Surface-plasmon-based optical manipulation. Laser Photonics Rev. **2**, 47 (2008).

[10] R. Zhao, P. Tassin, T. Koschny & C. M. Soukoulis, Optical forces in nanowire pairs and metamaterials. Opt. Express **18**, 25665 (2010).

[11] Miljkovic, V. D., Pakizeh, T., Sepulveda, B., Johansson, P. & Kall, M. Optical Forces in Plasmonic Nanoparticle Dimers. *The Journal of Physical Chemistry C* **114,** 7472 (2010).
